# Supplementary material for: Global covariation of forest age transitions with the net carbon balance
Source: Nat Ecol Evol. 2025 Aug 19;9(10):1848–60. doi: 10.1038/s41559-025-02821-5 (PMC12507667; doi:10.1038/s41559-025-02821-5)
Supplement: Supplementary file 1 — Supplementary Tables 1–5 and Figs. 1–8. [file 41559_2025_2821_MOESM1_ESM.pdf]

nature ecology & evolution

Supplementary information https://doi.org/10.1038/s41559-025-02821-5

Global covariation of forest age transitions with the net carbon balance

In the format provided by the authors and unedited

Supplementary materials

Table S1. Area-weighted forest age for 2010 and 2020 across the eleven TRANSCOM-land regions (Fig. S3). The difference between the forest age in 2020 and 2010 is also reported. The median values and quantiles at the 5th and 95th percentiles are reported across the 20 members.

Region	Forest age 2010	Forest age 2020	Forest age difference
**Eurasia Boreal**	$107.95^{116.53}_{88.98}$	$113.61^{121.98}_{95.09}$	$+5.67^{+6.16}_{+5.41}$
**NA Boreal**	$101.61^{111.86}_{85.67}$	$110.50^{120.69}_{94.61}$	$+8.90^{+9.04}_{+8.84}$
**Eurasia Temperate**	$44.51^{53.17}_{36.54}$	$52.49^{60.39}_{42.92}$	$+7.67^{+8.21}_{+6.38}$
**Europe**	$81.50^{91.19}_{72.08}$	$89.50^{98.91}_{80.28}$	$+7.97^{+8.20}_{+7.71}$
**NA temperate**	$85.72^{90.07}_{75.64}$	$92.28^{96.50}_{82.06}$	$+6.48^{+6.81}_{+6.24}$
**SA Temperate**	$87.38^{110.14}_{51.64}$	$91.04^{110.48}_{57.70}$	$+3.85^{+6.26}_{+1.16}$
**SA tropical**	$251.92^{260.24}_{237.23}$	$247.10^{254.32}_{233.88}$	$-4.67^{-3.41}_{-6.24}$
**Tropical Asia**	$139.43^{152.45}_{121.16}$	$132.41^{142.29}_{116.38}$	$-7.31^{-4.98}_{-10.04}$
**Northern Africa**	$113.42^{130.99}_{95.68}$	$110.93^{127.17}_{95.71}$	$-1.41^{+0.99}_{-4.99}$
**Southern Africa**	$105.22^{111.46}_{93.85}$	$106.89^{112.00}_{96.51}$	$+2.23^{+3.09}_{+0.30}$
**Australia**	$67.66^{87.98}_{55.82}$	$69.85^{87.67}_{57.86}$	$+2.07^{+3.40}_{-0.31}$

Table S2. Total NEE for 2010 and 2020 across TRANSCOM-Land regions. NEE changes between 2020 and 2010 have also been reported. The estimates are expressed in PgC year^{-1}. The median values and the 5th and 95th quantiles across the GCB2023 members are reported. For total NEE, a positive sign means a carbon source, while a negative sign means a decrease in carbon sink or an increase in carbon source for NEE changes and vice-versa. A forest mask was applied before doing the area-weighted total estimates.

Region	NEE 2010	NEE 2020	NEE changes
**Australia**	$-0.18^{-0.072}_{-0.38}$	$-0.12^{+0.020}_{-0.21}$	$+0.11^{+0.23}_{-0.026}$
**Eurasia Boreal**	$-0.56^{-0.32}_{-0.85}$	$-0.46^{-0.19}_{-0.90}$	$+0.065^{+0.25}_{-0.12}$
**Eurasia Temperate**	$-0.76^{-0.27}_{-1.35}$	$-0.62^{-0.20}_{-1.36}$	$+0.019^{+0.35}_{-0.17}$
**Europe**	$-0.66^{-0.17}_{-1.03}$	$-0.45^{-0.058}_{-0.78}$	$+0.061^{+0.52}_{-0.14}$
**NA Boreal**	$-0.36^{-0.11}_{-0.53}$	$-0.33^{-0.24}_{-0.48}$	$+0.0011^{+0.10}_{-0.14}$
**NA Temperate**	$-0.43^{+0.054}_{-0.90}$	$-0.72^{+0.10}_{-1.098}$	$-0.084^{+0.11}_{-0.50}$
**Northern Africa**	$-0.053^{+0.23}_{-0.32}$	$-0.059^{+0.37}_{-0.26}$	$+0.0098^{+0.26}_{-0.22}$
**SA Temperate**	$-0.21^{+0.20}_{-0.60}$	$-0.31^{+0.32}_{-0.41}$	$+0.063^{+0.26}_{-0.27}$
**SA Tropical**	$-0.20^{+0.30}_{-0.36}$	$+0.051^{+0.27}_{-0.47}$	$+0.064^{+0.56}_{-0.30}$
**Southern Africa**	$-0.075^{+0.15}_{-0.29}$	$+0.097^{+0.31}_{-0.20}$	$+0.15^{+0.43}_{-0.033}$
**Tropical Asia**	$-0.27^{+0.11}_{-0.90}$	$-0.26^{+0.063}_{-0.90}$	$-0.023^{+0.023}_{-0.17}$
**Global**	$-4.22^{-2.72}_{-4.65}$	$-3.51^{-2.18}_{-4.61}$	$+0.38^{+0.86}_{+0.030}$

Table S3. Regional fraction of undisturbed ageing and stand-replaced forests and their ratio. Fraction estimates are relative to the total area of undisturbed ageing and stand-replaced forests. Total area estimates are shown in brackets and are expressed in billions of hectares. The median values and quantiles at the 5th and 95th percentiles are reported across the 20 members.

**Region**	**Fraction of undisturbed ageing**	**Fraction of stand-replaced**	**Area ratio**
**Eurasia Boreal**	$0.17_{0.17}^{0.17}$ $(0.54_{0.54}^{0.54})$	$0.087_{0.070}^{0.11}$ $(0.020_{0.019}^{0.020})$	$0.036_{0.036}^{0037}$
**NA Boreal**	$0.069_{0.068}^{0.071}$ $(0.22_{0.21}^{0.22})$	$0.0099_{0.0078}^{0.013}$ $(0.0022_{0.0022}^{0.0023})$	$0.010_{0.010}^{0.010}$
**Eurasia Temperate**	$0.061_{0.057}^{0.062}$ $(0.19_{0.18}^{0.20})$	$0.084_{0.070}^{0.11}$ $(0.018_{0.013}^{0.033})$	$0.093_{0.067}^{0.19}$
**Europe**	$0.10_{0.10}^{0.10}$ $(0.32_{0.32}^{0.32})$	$0.029_{0.024}^{0.040}$ $(0.0065_{0.064}^{0.075})$	$0.020_{0.019}^{0.023}$
**NA temperate**	$0.092_{0.090}^{0.092}$ $(0.29_{0.28}^{0.29})$	$0.054_{0.049}^{0.071}$ $(0.012_{0.011}^{0.016})$	$0.042_{0.039}^{0.055}$
**SA Temperate**	$0.042_{0.041}^{0.043}$ $(0.13_{0.13}^{0.13})$	$0.037_{0.030}^{0.054}$ $(0.0088_{0.0063}^{0.011})$	$0.067_{0.047}^{0.084}$
**SA tropical**	$0.20_{0.20}^{0.21}$ $(0.64_{0.63}^{0.64})$	$0.11_{0.10}^{0.13}$ $(0.026_{0.020}^{0.030})$	$0.041_{0.031}^{0.047}$
**Tropical Asia**	$0.089_{0.088}^{0.094}$ $(0.28_{0.27}^{0.30})$	$0.26_{0.22}^{0.28}$ $(0.059_{0.040}^{0.070})$	$0.21_{0.13}^{0.26}$
**Northern Africa**	$0.079_{0.074}^{0.087}$ $(0.26_{0.24}^{0.30})$	$0.22_{0.11}^{0.25}$ $(0.048_{0.018}^{0.067})$	$0.19_{0.064}^{0.29}$
**Southern Africa**	$0.077_{0.074}^{0.080}$ $(0.24_{0.23}^{0.25})$	$0.10_{0.068}^{0.13}$ $(0.022_{0.014}^{0.035})$	$0.095_{0.055}^{0.15}$
**Australia**	$0.012_{0.011}^{0.012}$ $(0.037_{0.035}^{0.038})$	$0.020_{0.017}^{0.024}$ $(0.0046_{0.0033}^{0.0058})$	$0.13_{0.087}^{0.16}$

Table S4. The total area of undisturbed ageing and stand-replaced forests per age class. The median values and the 5th and 95th quantiles across the 20 members are reported.

	Young forests (0-20 years)	Maturing forests (21-80 years)	Mature forests (81-200 years)	Old forests (>200 years)
**Undisturbed ageing forests**	$0.61_{0.54}^{0.66}$	$0.60_{0.52}^{0.66}$	$0.98_{0.83}^{1.06}$	$1.027_{0.96}^{1.083}$
**Stand-replaced forests**	$0.14_{0.083}^{0.20}$	$0.029_{0.022}^{0.037}$	$0.034_{0.031}^{0.036}$	$0.033_{0.028}^{0.041}$

Table S5. Total carbon stocks and changes of undisturbed ageing and stand-replaced forests across age classes. Total estimates are expressed in PgC, while the stock changes are described in PgC year^{-1}. The median values and quantiles at the 5th and 95th percentiles are reported across the 20 members.

	Young forests (0-20 years)	Maturing forests (21-80 years)	Mature forests (81-200 years)	Old forests (>200 years)	All age classes
**AGC undisturbed ageing forests**	$17.99_{16.02}^{21.32}$	$31.29_{26.40}^{35.68}$	$60.51_{47.32}^{79.34}$	$127.39_{99.20}^{155.14}$	$235.75_{192.50}^{286.11}$
**AGC stand-replaced forests**	$1.85_{1.35}^{3.17}$	$1.00_{0.86}^{1.13}$	$1.58_{1.26}^{2.03}$	$2.18_{1.58}^{2.59}$	$6.83_{5.78}^{7.86}$
**AGC changes stand-replaced forests**	$+0.061_{+0.027}^{+0.10}$	$+0.099_{+0.078}^{+0.15}$	$+0.096_{+0.090}^{+0.10}$	$+0.14_{+0.10}^{+0.16}$	$+0.38_{+0.35}^{+0.46}$

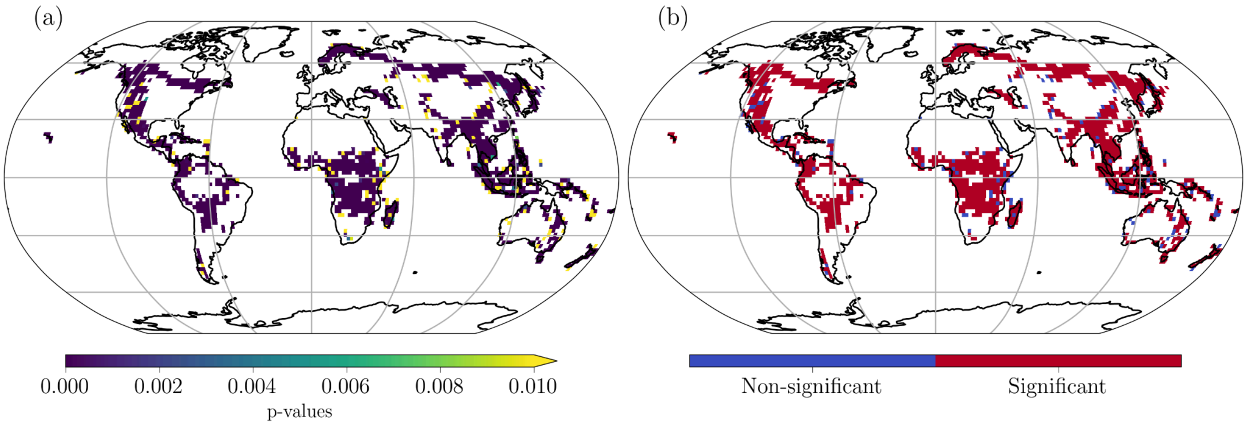

Fig. S1. Significant level of the role of management type on the relationship between forest age and biomass (see methods) within a 2-degree × 2-degree spatial window.

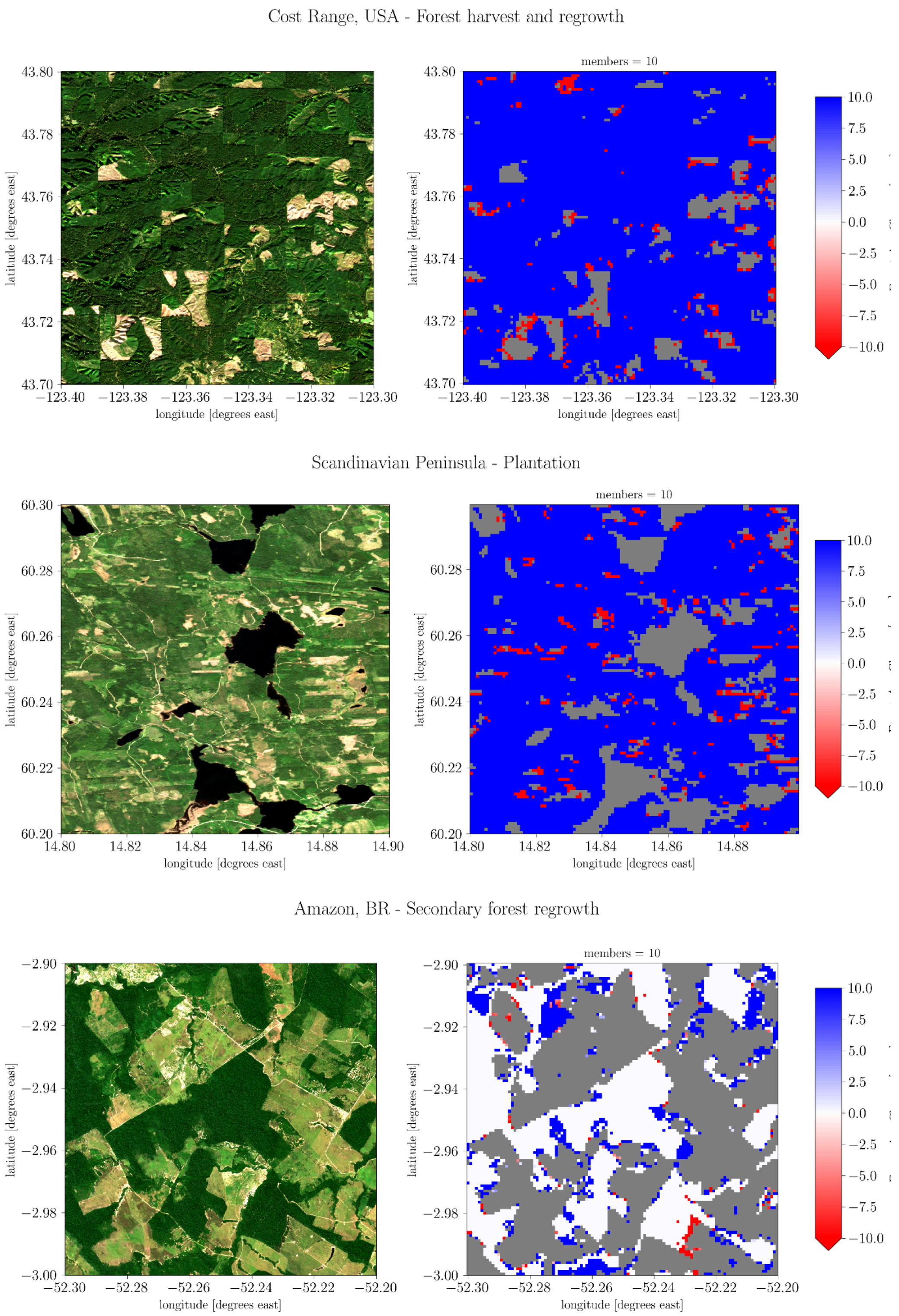

Fig. S2. The GAMIv2.0 forest age product at native resolution (i.e., 100m pixel size) provides a detailed view of substantial changes in forest age between 2010 and 2020, enhancing the precision of our analysis.

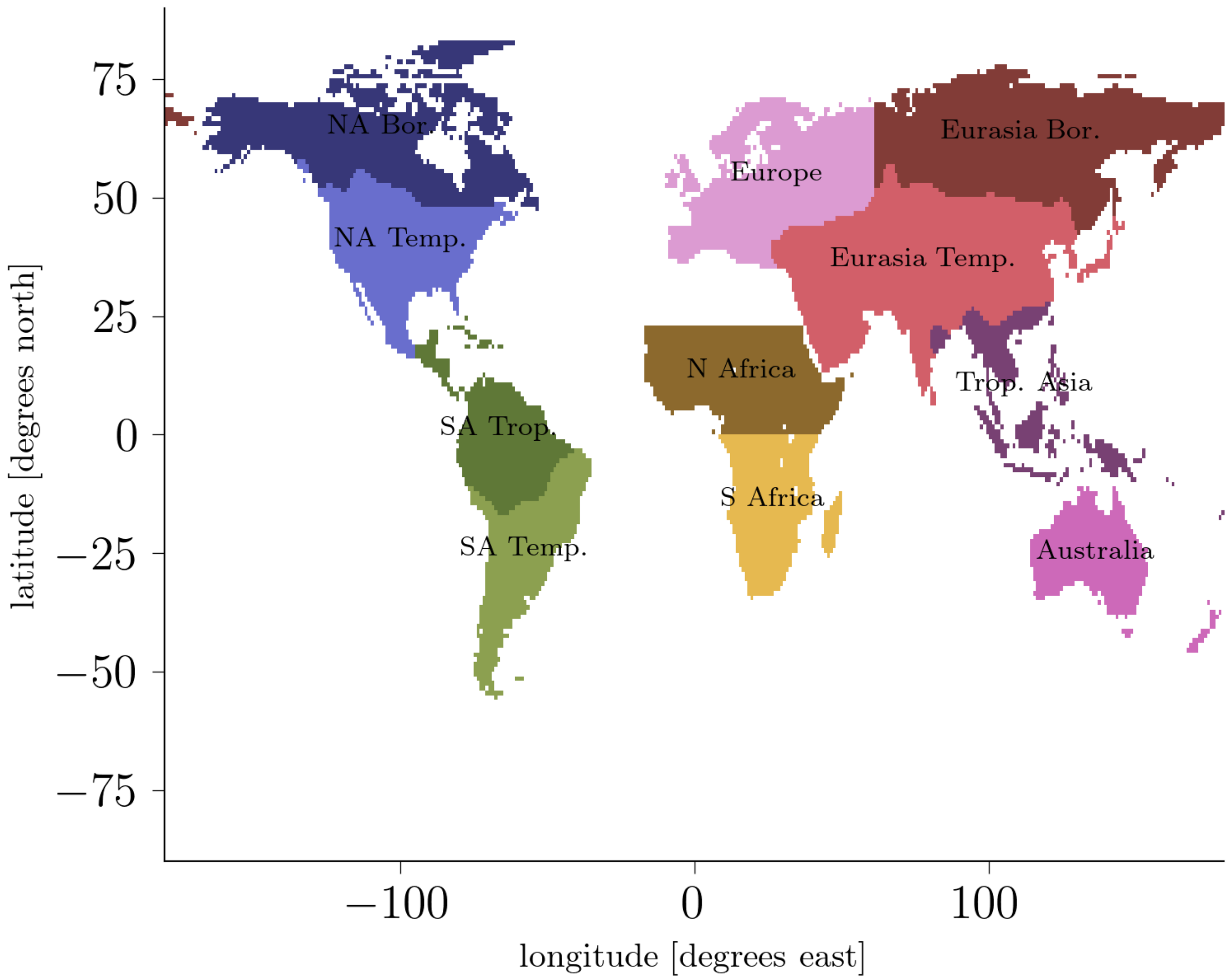

Fig. S3. Spatial distribution of the eleven TRANSCOM-land regions. NA: North America, SA: South America, N: Northern, S: Southern, Temp.: Temperate, Bor.: Boreal, Trop.: Tropical.

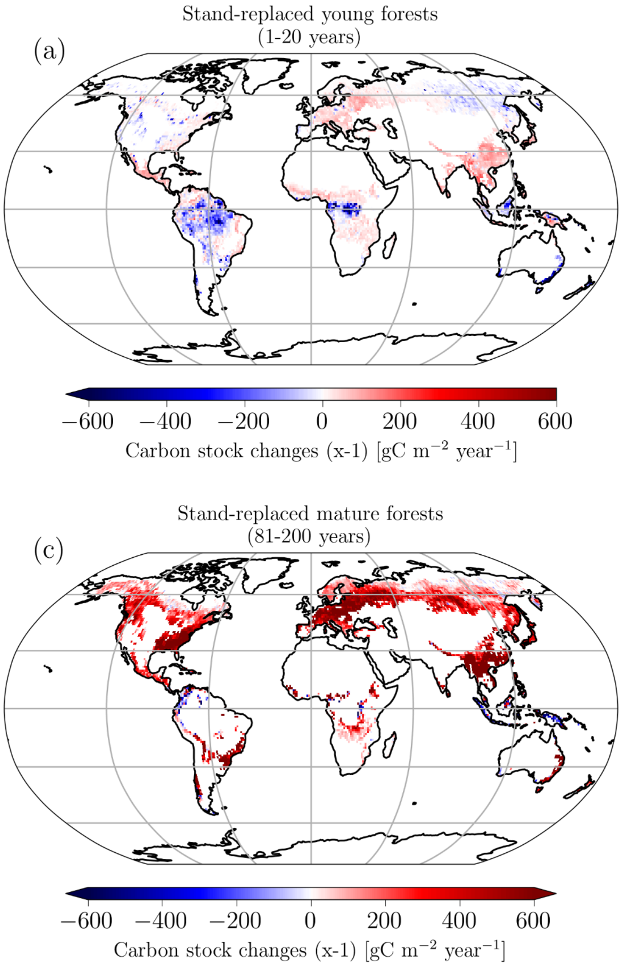

Fig S4. Spatial distribution of carbon stock changes (in gC m^{-2} $year^{-1}$) in stand-replaced forests categorised by age before stand-replacement: (a) young (≤ 20 years), (b) intermediate (21–80 years), (c) mature (81–200 years), and (d) old (> 200 years) in 2010. Each pixel represents the median carbon stock change for 100m resolution pixels belonging to a specific forest age category, aggregated at a 1-degree scale. Positive values indicate carbon loss (biosphere-to-atmosphere flux) to maintain consistency with flux sign conventions, while negative values indicate carbon accumulation.

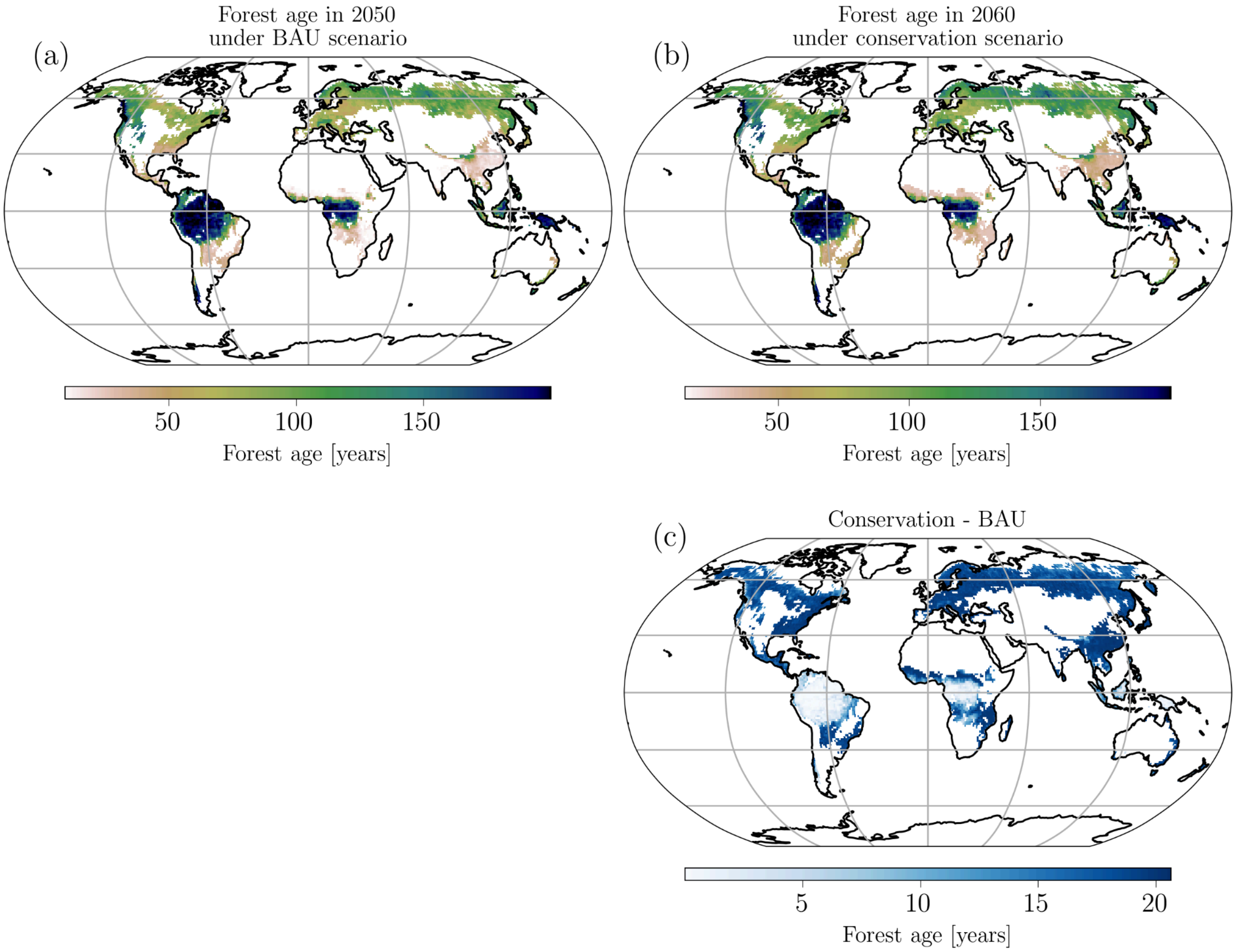

Fig. S5. Weighted-area average forest age for the (a) BAU and (b) forest conservation scenarios in 2050. The (c) difference between the forest age maps is also shown.

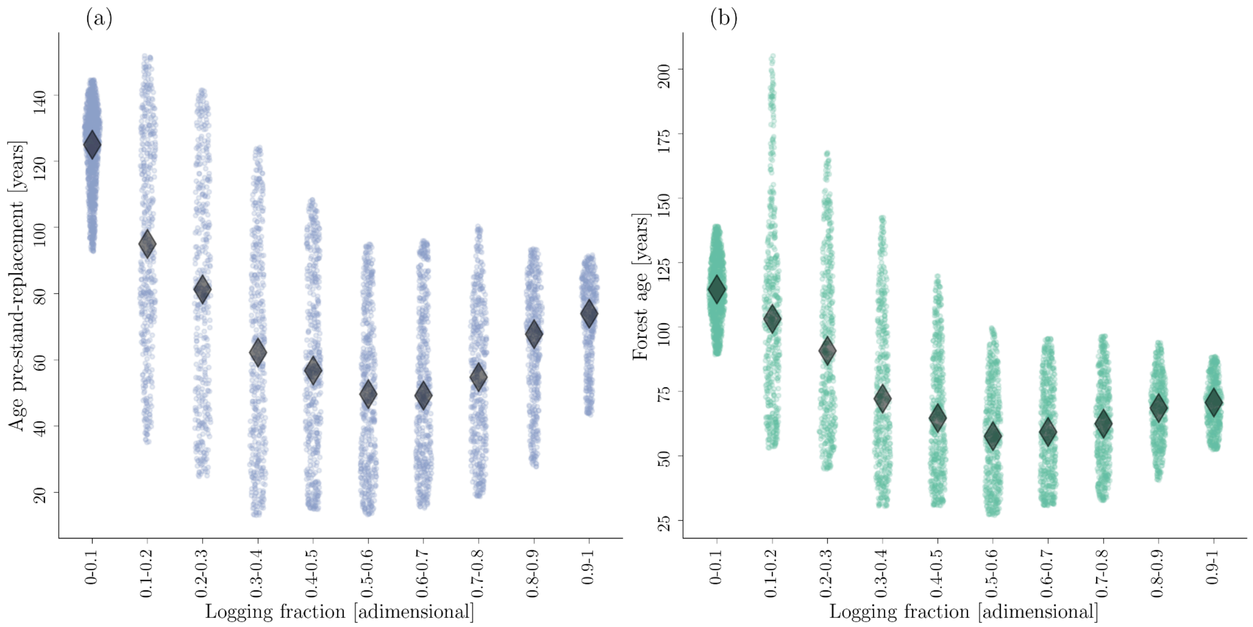

Fig. S6. Logging fraction against age pre-stand-replacement (a) and forest age (b).

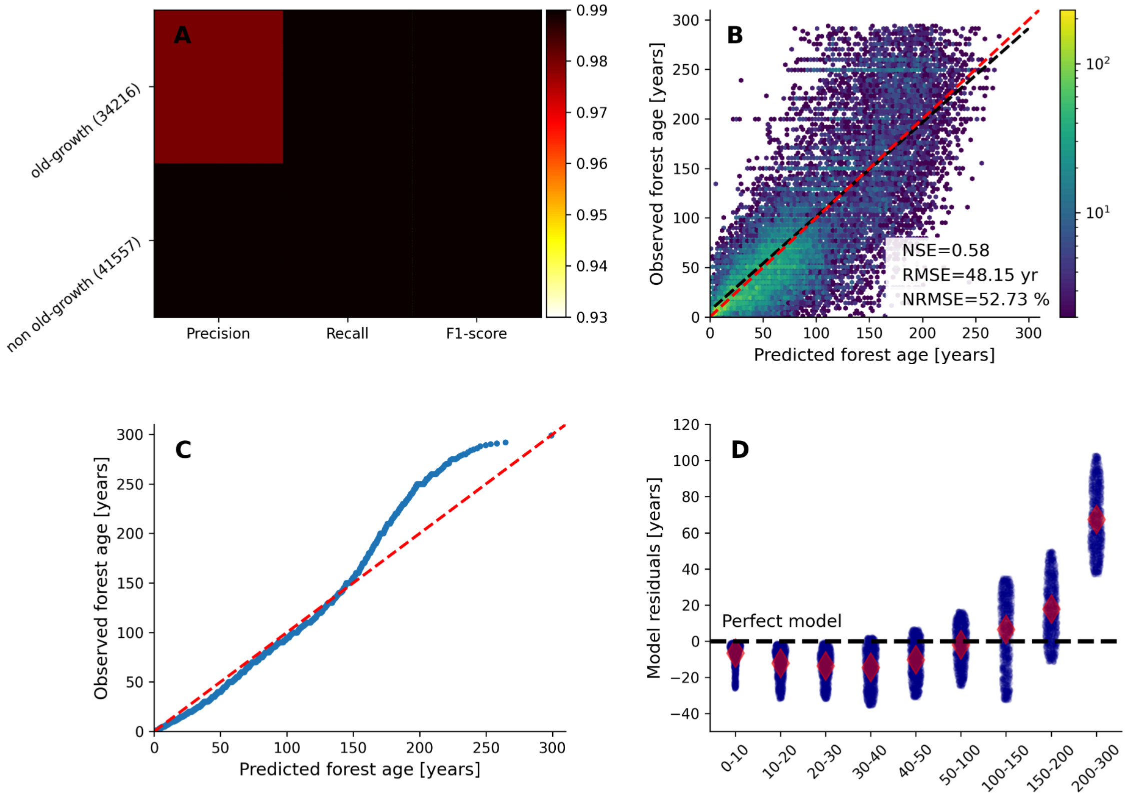

Fig. S7. Cross-validated results of the old-forest vs. non-old-forest classification (a) and comparison of predicted vs. observed forest age estimates from the regression model (b). The quantile-quantile plot (c) and the model residuals across age classes (d) are also shown.

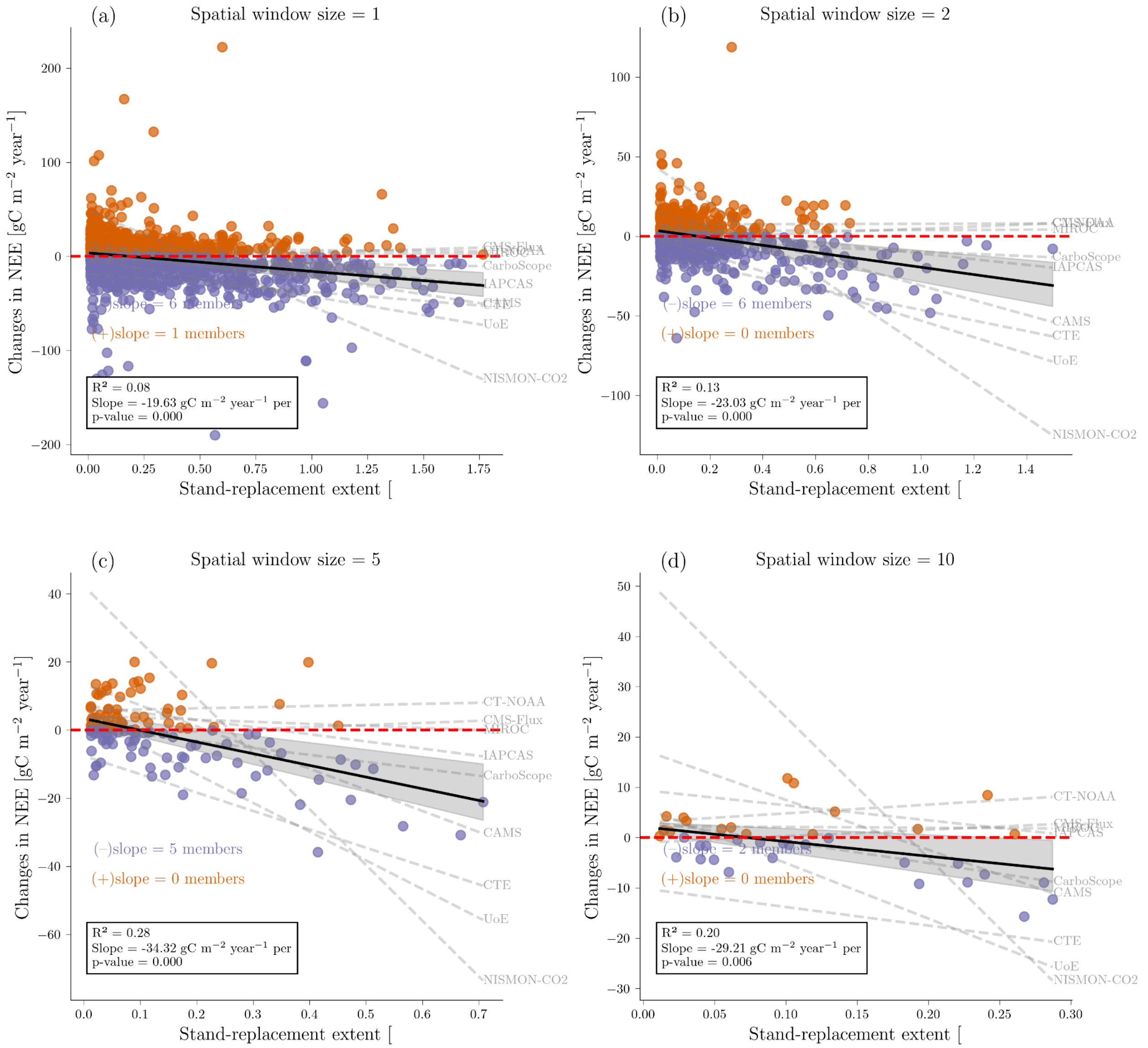

Fig S8. Relationship between the fraction of old forests replaced by young stands (i.e., stand-replacement extent) and changes in net CO_2 fluxes between circa 2020 (average of 2019–2021) and 2010 (average of 2009–2011) using different spatial windows: none (a), 2x2 degree (b), 5x5 degree (c) and 10x10 degree (d). The dark solid line represents the linear regression on the ensemble estimates, while the dashed grey lines indicate the regressions for the nine individual atmospheric inversion models. To smooth the spatial distribution of net CO_2 fluxes, we applied a Gaussian filter (length = 500 km, equivalent to approximately four one-degree pixels). This smoothing technique reduces noise in the data and helps minimise the influence of local transport errors.
